# Construction of Flexible Deterministic Sparse Measurement Matrix in Compressed Sensing Using Legendre Sequences

**DOI:** 10.3390/s24227406

**Published:** 2024-11-20

**Authors:** Haiqiang Liu, Ming Li, Caiping Hu

**Affiliations:** 1 School of Information and Control Engineering, China University of Mining and Technology, Xuzhou 221116, China; 00000005207@jit.edu.cn; 2 School of Computer Engineering, Jinling Institute of Technology, Nanjing 211169, China; hucp@jit.edu.cn; 3 CCTEG Changzhou Research Institute, Changzhou 223000, China

**Keywords:** compressed sensing, deterministic binary measurement matrix, Legendre sequence, phase transition

## Abstract

Compressed sensing (CS) is an innovative signal acquisition and reconstruction technology that has broken through the limit of the Nyquist sampling theory. It is widely employed to optimize the measurement processes in various applications. One of the core challenges of CS is the construction of a measurement matrix. However, traditional random measurement matrices are often impractical. Additionally, many existing deterministic binary measurement matrices fail to provide the required flexibility for practical applications. In this study, inspired by the observation that pseudo-random sequences share similar properties with random sequences, we constructed a deterministic sparse measurement matrix with a flexible measurement number based on an pseudo-random sequence—the Legendre sequence. Empirical analysis of the phase transition and an assessment of the practical features of the proposed measurement matrix were conducted. We validated the effectiveness of the proposed measurement matrix on randomly synthesized signals and images. The results of our simulations reveal that our proposed measurement matrix performs better than several other measurement matrices, particularly in terms of accuracy and efficiency.

## 1. Introduction

In numerous applications of wireless sensor networks (WSNs), such as wireless body sensor networks (WBSNs) [1], micro-seismic monitoring wireless sensor networks (MMWSNs) [2], and wireless video monitoring systems (WVMSs) [3], sensors face limitations in energy, bandwidth, computational capability, and storage space. To sustain the lifespan of WSNs and conserve their bandwidth, it is crucial to reduce the amount of data transmitted. Compressed sensing (CS) allows for the acquisition of fewer measurements directly at the sensor, significantly reducing the amount of data to be transmitted.

Compressed sensing (CS) [4] is a novel signal acquisition and reconstruction technology that has broken through the limit of the Nyquist sampling theory. By utilizing sparsity, CS allows signals to be sampled at rates significantly below the Nyquist rate by projecting them onto a measurement matrix. The original signal can then be efficiently recovered by solving an optimization problem based on the sparse representation of the signal. CS transfers the computational burden from the encoder to the decoder, which is particularly suitable for sensors with constrained computational capabilities. Thanks to these advantages, CS is extensively employed to optimize the measurement process in diverse applications, including synthetic aperture radar (SAR), wireless body area networks [1], the health monitoring of pipelines [5], micro-seismic monitoring [2], and magnetic resonance imaging [6].

One of the core issues of CS is the measurement process. In sensors, the data are projected onto a measurement matrix to reduce the number of required measurements. Considering the limitations in the computational capability and storage space of sensors, the measurement matrix must be simple and efficient. Many measurement matrices have been proposed, primarily falling into two categories: random measurement matrices and deterministic measurement matrices.

Initially, random measurement matrices with elements following independent identical distribution (IID) [7] or Bernoulli distribution [8] were proposed. Having been proven to follow the restricted isometry property (RIP) [8] with high probability, they have been extensively researched. Inspired by the fact that symmetric sign ensembles offer computational advantages over Gaussian ensembles, Zhang G. et al. [9] proposed a measurement matrix based on the symmetric sign ensemble and demonstrated that the random sparse measurement matrix follows the RIP with high probability. Although random measurement matrices have good properties, they are often impractical. Furthermore, as they cannot be reproduced, they must be transmitted from the encoder to the decoder and require a large amount of storage space, which is impractical.

Deterministic measurement matrices can overcome the limitations of random measurement matrices. Specifically, deterministic sparse measurement matrices consist of only two elements: ‘0’ and ‘1’. They have low computational complexity and require minimal storage space, making them particularly suitable for resource-restrained sensors. Numerous deterministic sparse measurement matrices have been proposed. DeVore R. A. [10] proposed a deterministic sparse measurement matrix using finite fields, but its size is constrained to $p^{2}\times p^{r+1}$ where p is a prime number and r is an integer. Li S. et al. [11] constructed several types of deterministic measurement matrices based on finite geometry; however, their dimensions are restricted to specific size of ${(q}^{3}$+$q^{2}+q+1)*[(q^{2}+1)(q^{2}+q+1)$], $q^{3}*{[q}^{2}(q^{2}+q+1)]$, ${(q}^{3}+1)*\left[q^{2}\left(q^{2}-q+1\right)\right],$ and ${(q}^{2}+1)*[q^{2}(q^{2}+1)]$ where q is a prime power. Xia S. T. et al. [12] and Tong F. H. et al. [13] constructed deterministic sparse measurement matrices based on finite geometry and unitary geometry. However, similar to the matrices created by Li S. et al. [11], their dimensions are likewise constrained. Naidu R. R. et al. [14] proposed a deterministic sparse measurement matrix based on Euler’s square; however, its size is constrained to $M\times c{\left(M\mu \right)}^{2}$ where M represents the measurement number, μ represents the mutual coherence, and $c\in \left[1,\left.2\right)\right.$. Lu C. et al. [15] constructed the deterministic bipolar measurement matrix utilizing pseudo-random sequences, utilizing pseudo-random sequences; however, the number of measurements is constrained to ${\;2}^{r}-1$ where r is an integer. Zhang G. et al. [16] proposed a more flexible deterministic bipolar measurement matrix based on Legendre sequences; however, the number of measurements is constrained to prime integer or even integer. Lu W. et al. [17] proposed a deterministic sparse measurement matrix with a flexible measurement number using the bipartite graph. However, based on our experimental results, its performance still requires further improvement. There are also many other deterministic sparse measurement matrices based on coding theory [18,19], expander graphs [20], balanced incomplete block designs [21], etc. However, due to the specific construction methods of deterministic sparse measurement matrices, their dimensions are often constrained to certain values, highlighting the need for more flexible designs.

Inspired by [9] and the observation that pseudo-random sequences share similar properties with random sequences, we constructed a deterministic sparse measurement matrix using an pseudo-random sequence known as the Legendre sequence.

The primary contributions of this paper include the following:

(1) A simple deterministic sparse measurement matrix is proposed utilizing the Legendre sequence. The proposed measurement matrix has a flexible measurement number, exhibits low computational capability, and requires small storage space.
(2) Empirical analysis of the phase transition is carried out and an evaluation of the practical features of the proposed measurement matrix is performed.
(3) The performance of the proposed measurement matrix is compared with that of several state-of-the-art measurement matrices using random signals and images.

This paper is organized as follows: In Section 2, we review the theories of CS and the Legendre sequence. Section 3 introduces the methodology used for constructing the measurement matrix. Additionally, the phase transitions and practical features of the proposed measurement matrix are analyzed. In Section 4, experiments on random signals and images are conducted. Finally, in Section 5, the conclusion is presented.

## 2. Background

### 2.1. Theory of CS

The underlying assumption in this paper is that the signal $x\in R^{N}$ itself is naturally sparse. That means than $x\in R^{N}$ has k≪N non-zero elements naturally.

There are mainly two steps in CS: the measurement process and the recovery process. In the measurement process, x is measured by projecting it onto the measurement matrix Φ, as shown in Equation (1):

(1) y=Φx

Since Φ is a matrix with a size of M×N, where M<N, x is projected into a lower-dimensional space. Clearly, the measurement ratio is M/N.

In the recovery process, x needs to be reconstructed from y by solving Equation (1). However, because M<N, this process becomes ill-posed. According to compressed sensing theory, if x is sparse, it can be approximately reconstructed by solving the following $l_{0}-$ optimization problem (2):

(2) $$min{\left\|x\right\|}_{0}\;\;s.t.\;y=\Phi x$$

However, this problem is NP-hard, thus a greedy algorithm, such as orthogonal matching pursuit (OMP) [22] can be used to find a good-enough solution. Donaho et al. [4] demonstrated that if the measurement matrix satisfies the RIP under a specific condition, problem (2) can be replaced with the $l_{1}-$ optimization problem (3), which can be solved efficiently using the basis pursuit (BP) algorithm [23].

(3) $$min{\left\|x\right\|}_{1}\;\;s.t.\;y=\Phi x$$

The RIP is an important criterion to verify whether Φ is a proper measurement matrix.

**Definition 1.**  *For a measurement matrix,* $\Phi \in R^{M\times N}$*, and any k-sparse signal, if there exists a constant $\delta_{s}\in \left(0,1\right)$, such that*

(4) $$\left(1-\delta_{s}\right){\left\|x\right\|}_{2}^{2}\leq {\left\|\Phi x\right\|}_{2}^{2}\leq \left(1+\delta_{s}\right){\left\|x\right\|}_{2}^{2}$$

*then,* Φ *is said to satisfy the RIP with order* k*. And the smallest number* $\delta_{k}$* is called the restricted isometry constant (RIC) of order* k.

We can evaluate whether a matrix is a good measurement matrix by checking whether it satisfies the condition (4) for a large k. However, RIP merely provides a sufficient condition to guarantee the accurate recovery of signals, which is very conservative in practical applications.

**Figure 1:**
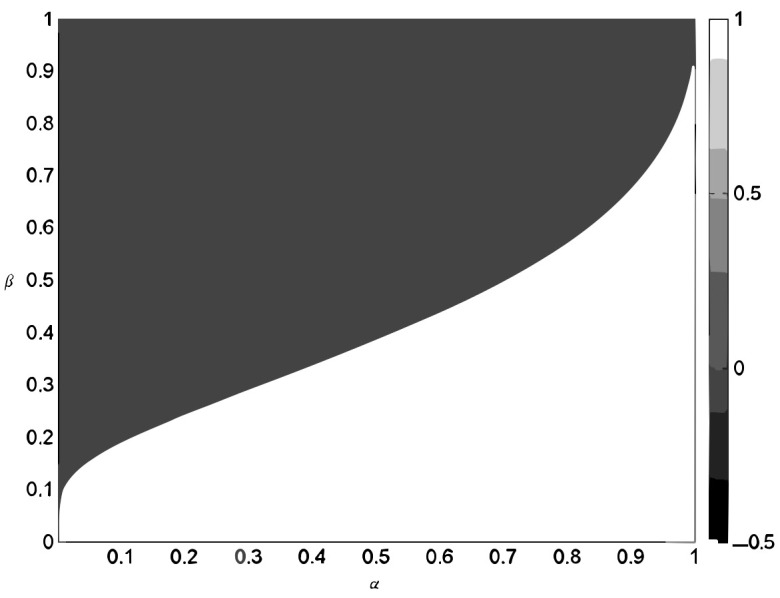
Phase diagram for $l_{1}$ minimization. Dark region: reconstruction success probability above 50%. Light region: reconstruction success probability below 50%.

Phase transition is more precise, serving as both a necessary and sufficiency condition. It is widely used to evaluate the effectiveness of measurement matrices [24] and recovery algorithms [25,26]. Let α=M/N represent the sampling ratio and β=k/N denote the sparsity ratio. It was observed that the plane of (α,β) can be partitioned into two distinct phases, as shown by the significant phase transition curve during signal recovery, as shown in Figure 1. In one phase, the original signal can be perfectly recovered, and in the other phase, recovery fails, with equally high probability. Phase transition is an important criterion and has garnered extensive research attention. The phase transition threshold for the Gaussian random measurement matrix was investigated in [27]. Numerical experiments demonstrated a nearly perfect alignment between the empirical 50% success curve and the theoretical bound [27].

### 2.2. Theory of Legendre Sequence

The research indicates that pseudo-random sequences exhibit similar properties to random sequences and can serve as substitutes in numerous scenarios. Benefiting from this, pseudo-random sequences are widely used in spectrum communication [28], data encryption [29], and many other fields [30]. The Legendre sequence has a more flexible period compared to other pseudo-random sequences.

Let p > 2 be a prime number, and a be a coprime integer of p; if the congruence

(5) $$s^{2}={\left(a\right)}_{p}$$

has an integer solution s, then a is referred to as a quadratic residue of modp; otherwise, it is termed a quadratic non-residue modulo p.

We define the Legendre symbol as

(6) $$\left(\frac{a}{p}\right)=\left\{\begin{array}{c} 0,\;\;\;a\;is\;quadratic\;residue\;of\;mod\;p\;\\ \;\;\;\;+1,\;\;\;a\;is\;quadratic\;non-residue\;of\;mod\;p\;\;\; \end{array}\right.$$

For any integer g, it follows

(7) $$\left(\frac{gp}{p}\right)=0$$

and

(8) $$\left(\frac{0}{p}\right)=0$$

Consequently, we can derive a Legendre sequence as

(9) $$\left(\frac{0}{p}\right),\left(\frac{1}{p}\right),\left(\frac{2}{p}\right),$$

It has been proven that when p is a prime number in the form of 4t−1 (where t is a positive integer), the Legendre sequence with a period of p is a pseudo-random sequence [31]. It is necessary to note that there are significantly more prime numbers in the form of 4t−1 compared to integers in the form of $2^{r}-1$(where r is a positive integer), which represents the periods of most other pseudo-random sequences. For example, the periods of m-sequences between 100 and 1000 are limited to 127, 255, and 511, while the Legendre sequences, in contrast, offer a notably flexible selection of periods, as shown in Table 1.

## 3. Proposed Method

### 3.1. Construction of Proposed Measurement Matrix

Our measurement matrix is applicable to signals of length N=4t−1, where t is a positive integer and N is a prime number. The construction method is as follows:

1. Let p=N, the following numbers are obtained: ${\left(1^{2}\right)}_{p}$,${\left(2^{2}\right)}_{p}$,$\cdots ,{\left({\left(\frac{p-1}{2}\right)}^{2}\right)}_{p}$.
2. If i=1,2,⋯,p−1 appears among the numbers obtained in step 1. Let $s_{i}=0$; otherwise, let $s_{i}=+1$.
3. Let $s_{0}=0$; then, a Legendre sequence, $S=\left\{s_{0},s_{1},\cdots ,s_{p-1}\right\}$, can be obtained.
4. A p×p matrix Q can be constructed using (10):
  (10) $$Q=\left[\begin{array}{ccccc} s_{0} & s_{1} & \cdots & s_{p-2} & s_{p-1}\\ s_{1} & s_{2} & \cdots & s_{p-1} & s_{0}\\ \vdots & \vdots & \ddots & \vdots & \vdots \\ s_{p-2} & s_{p-1} & \cdots & s_{p-4} & s_{p-3}\\ s_{p-1} & s_{0} & \cdots & s_{p-3} & s_{p-2} \end{array}\right]$$
5. The top M,(1<M<p) rows from matrix Q are selected. Then, a M×p deterministic sparse measurement matrix is obtained. According to compressed sensing theory, when M exceeds a certain threshold, the signal can be reconstructed with high probability. Therefore, M can be set based on the sparsity of the signal. Typically, M is required to satisfy the condition M>Cklog(N/k), where N is the signal dimension and C is a constant related to the desired reconstruction accuracy.

### 3.2. Analysis of Phase Transition

We analyzed the empirical phase transition of the proposed measurement matrix and compared it with several state-of-the-art alternatives. It is worth noting that for deterministic sparse measurement matrices with inflexible measurement numbers, analysis of their phase transitions becomes unfeasible. The compared measurement matrices included the Gaussian random measurement matrix [7], the random symmetric measurement matrix [9], the random binary measurement matrix [9], and the bipartite graph measurement matrix [17]. The latter is a deterministic sparse measurement matrix with a flexible measurement number, and the others are random measurement matrices.

The Gaussian random measurement matrix is a classic and highly effective measurement matrix. It is one of the most commonly used benchmarks in the study of measurement matrices due to its excellent performance. The random symmetric and random binary measurement matrices are classic matrices that share structural similarities with our proposed measurement matrix. Additionally, we included the bipartite graph measurement matrix in our comparisons due to the fact that it is a classic deterministic sparse matrix, known for its flexibility in dimensions and good sparsity properties.

In the Gaussian random measurement matrix, the entries are independently sampled from a Gaussian normal distribution with a mean of 0 and a variance of 1. In the random symmetric measurement matrix, the entries are independently sampled from a symmetric signs distribution, where each element takes the value of $+\frac{1}{\sqrt{N}}$ or $-\frac{1}{\sqrt{N}}$ with equal probability. In the random binary matrix, the entries are independently sampled from a distribution where each element takes the value of $+\frac{2}{\sqrt{N}}$ or 0 with equal probability. The bipartite graph measurement matrix, constructed using the Progressive Edge-Growth (PEG) algorithm, is a low-density parity-check (LDPC) matrix that generally contains d non-zero elements in each column, where d<<N.

Phase transition was achieved utilizing the methodology described in references [25,32,33], as follows. For a fixed N=239, α varied from 0.1 to 0.95 with a step size of 0.05, and k varied from 1 to M with a step size of 1. At each combination of (α,β), 300 sparse signals were measured using different measurement matrices and then recovered using the OMP algorithm [22]. Let x be the original signal and $\hat{x}$ be the recovered signal. For each trial, if

(11) $$\frac{{\left\|x-\hat{x}\right\|}_{2}}{{\left\|x\right\|}_{2}}\;<\;0.01$$

we declare that the recovery is successful. Then, for each (α,β)-tuple and each measurement matrix, we can obtain the exact recovery rate. Since it was observed that the empirical 50% success curve closely aligned with the theoretical bound [27], we employed the logistic regression method as outlined in references [25,32,33]. That is, for each α, the phase transition referred to the value of β that yielded a 50% exact recovery rate in this study.

For greater universality, empirical analysis of the phase transitions was conducted on various measurement matrices for two types of randomly synthesized sparse signals: Gaussian sparse signals and binary uniform sparse signals. The generation process was as follows: the non-zero elements of the Gaussian sparse signal followed a standard Gaussian distribution, whereas the non-zero elements of the binary uniform signal followed a uniform distribution in the range [0, 1].

**Figure 2:**
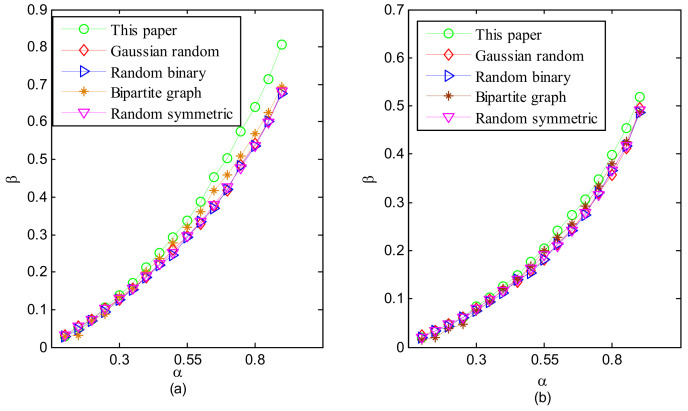
Phase transitions of several measurement matrices for (**a**) Gaussian sparse signals and (**b**) binary uniform sparse signals.

The empirical analysis results are depicted in Figure 2. Obviously, the Gaussian random measurement matrix, the random symmetric measurement matrix, and the random binary measurement matrix exhibited similar phase transition curves. And the deterministic bipartite graph measurement matrix demonstrated a superior phase transition curve compared to the aforementioned random measurement matrices in the cases of Gaussian sparse signals, while exhibiting similar phase transition curves for binary uniform sparse signals. Furthermore, our proposed measurement matrix outperformed all other measurement matrices for both Gaussian sparse signals and binary uniform sparse signals.

### 3.3. Analysis of Practical Features

The aforementioned phase transition property of the proposed measurement matrix ensures its effective ability to capture information. Nevertheless, in practical applications, certain practical features of measurement matrices must be taken into consideration, including computational complexity in the measurement process and memory cost. The practical features of the aforementioned measurement matrices are analyzed in Table 2. For fairness, we assumed that the size of the measurement matrix was M×N, and each floating-point element required B bits of storage space. Evidently, the proposed measurement matrix possesses the following advantages:

(1) A flexible number of measurements: The proposed measurement matrix provides a flexible number of measurements.
(2) Low computational complexity: As a sparse measurement matrix, the proposed matrix requires only addition operations during the measurement process, without involving multiplication. Compared to multiplication, addition operations are computationally less intensive, especially in binary matrices, where bit-wise addition is much simpler and less computationally expensive than multiplication. Therefore, this approach significantly reduces computational load.
(3) Low memory cost: The Gaussian random matrix, characterized by its floating-point entries, requires the largest storage space, specifically M×N× *B* bits. In contrast, for the symmetric measurement matrix and the random binary measurement matrix, since the two possible values can be encoded with just 1 bit, their storage space is significantly reduced to M×N bits. The bipartite graph measurement matrix requires $N\times d\times \log_{2}M$ bits, as it only stores the positions of the non-zero entries. The measurement matrix proposed in this paper only requires the storage of a single Legendre sequence, as all other columns can be generated by cyclically shifting this Legendre sequence, which requires merely N bits. This storage requirement is significantly lower compared to the other measurement matrices discussed.

**Table 2:** Computational complexities of various measurement matrices.

Φ	Randomness or Deterministic	Measurement-Flexible	Memory Cost (Bits)	Multiplierless
Gaussian random	Randomness	Yes	*BMN*	No
Random symmetric	Randomness	Yes	*MN*	No
Random binary	Randomness	Yes	*MN*	No
Bipartite graph	Deterministic	Yes	$N\times d\times \log_{2}M$	Yes
This paper	Deterministic	Yes	*N*	Yes

The aforementioned advantages make our proposed measurement matrix more suitable for practical applications, especially in scenarios involving resource-constrained sensors. In the following section, we compare the performance of our proposed measurement matrix with the above measurement matrices using random signals and images.

## 4. Experimental Results and Analysis

In this section, we compare the performance of our proposed measurement matrix with several existing ones: the Gaussian random measurement matrix [7], the random symmetric measurement matrix [9], the random binary measurement matrix [9], and the bipartite graph measurement matrix [17]. The phase transitions and practical characteristics of all of these measurement matrices were analyzed in Section 3. To ensure greater versatility, we employed two types of signals as test signals: randomly synthesized signals and images. The OMP algorithm [22] was used for signal recovery.

All experiments were conducted using Matlab R2014a on a computer equipped with an Intel Core i7-6700 CPU (3.4 GHz) and 16 GB of RAM, running Windows 10.

### 4.1. Random Synthesized Sparse Signals

In this part, two kinds of randomly generated sparse signals were used as test signals: Gaussian random sparse signals and binary uniform sparse signals. Their generation method was elaborated on in Section 3. In the noiseless scenario, the performance of the measurement matrix was assessed by the ratio between the number of successful reconstructions and the total number of trials. A successful reconstruction condition is defined in (11), and number of total trials was 300.

**Figure 3:**
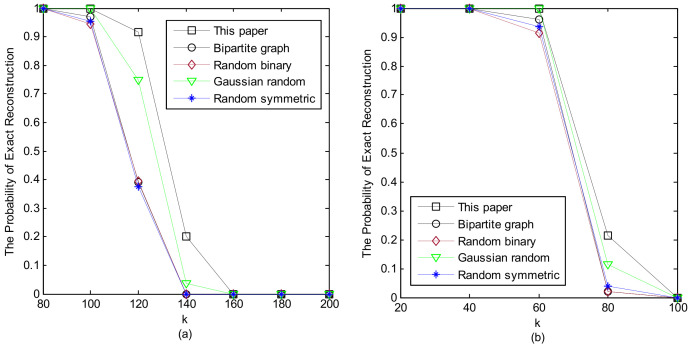
Exact recovery percentages of various measurement matrices against different values of *k* for (**a**) Gaussian random sparse signals and (**b**) uniform random sparse signals with M=263 and N=599.

**Figure 4:**
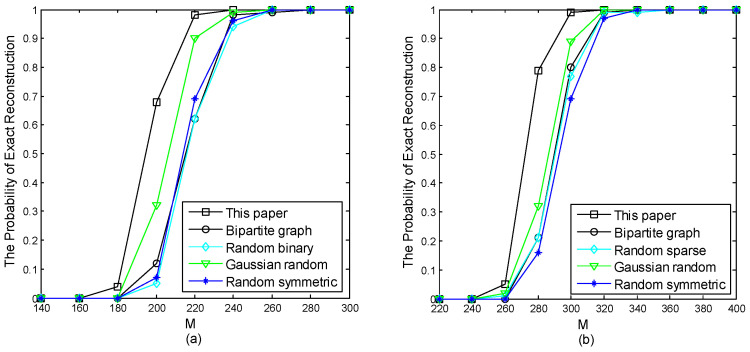
Exact recovery percentages of various measurement matrices for (**a**) Gaussian random sparse signals and (**b**) uniform random sparse signals with k=100 and N=431, against varying M.

Figure 3 depicts the perfect recovery percentages of different measurement matrices as sparsity *k* varies with M=263 and N=599. Figure 4 illustrates the perfect recovery percentages of various measurement matrices as M changes with k=100 and N=431. Our proposed measurement matrix demonstrated superior performance, particularly in practical applications due to its enhanced features.

In a noisy scenario, the process can be represented as

(12) y=Φx+e

where $e\in R^{M}\;$ represents Gaussian white noise. In this experiment, ${\left\|e\right\|}_{2}={0.1*\left\|y\right\|}_{2}$. SNR was used to evaluate the performance of different measurement matrices, which is defined as

(13) $$SNR=20\log_{10}\left(\frac{{\left\|x\right\|}_{2}}{{\left\|x-\hat{x}\right\|}_{2}}\right)$$

where x is the original signal, and $\hat{x}$ is the reconstructed signal.

The SNRs of different measurement matrices for Gaussian random sparse signals and uniform random sparse signals against changes in M with k=100 and N=431 are shown in Table 3. It can be seen that for both Gaussian random sparse signals and uniform random sparse signals, our proposed measurement matrix exhibited a higher SNR compared to the other measurement matrices. This suggests that our proposed measurement matrix exhibits greater robustness to noise compared to the others.

### 4.2. Two-Dimensional Image

**Figure 5:**
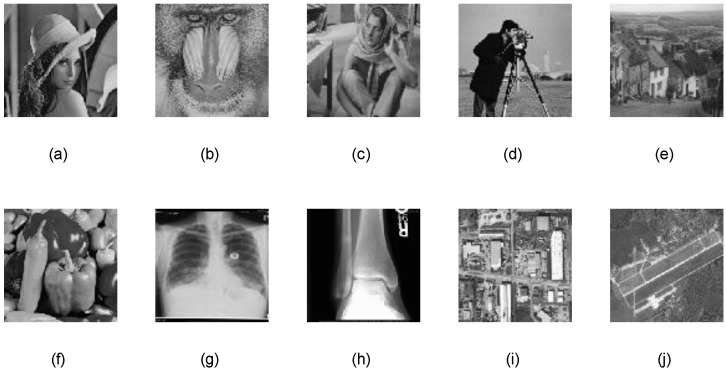
Test images: (**a**) Lena, (**b**) Baboon, (**c**) Barbara, (**d**) Cameraman, (**e**) Goldhill, (**f**) Peppers, (**g**) Bone1, (**h**) Bone2, (**i**) Aerial image1, and (**j**) Aerial image2.

In this part, we evaluate the performance of various measurement matrices for image reconstruction under compressed sensing. As shown in Figure 5, the tested images included six commonly used test images, two medical images, and two aerial images. The size of all images was 223*223. We utilized block-based compressive sensing to recover the tested images. As compressed sensing relies on sparsity, we achieved image sparsity by retaining a certain proportion of coefficients under the discrete Fourier transform. In this paper, this proportion was set to 30%.

In the block-based compressive sensing, the size of image sub-blocks was set as 223 × 1. This means that each column of the original image was treated as a sub-block. Each sub-block was compressed individually in the measurement process. In the recovery process, each image block was separately reconstructed using OMP and then assembled to reconstruct the complete image.

The peak signal-to-noise ratio (PSNR) was used to assess the quality of the recovered image. For two images of m×n, G, and $\hat{G}$, the PSNR was calculated using the following expression:

(14) $$PSNR=10\log_{10}\left(\frac{223^{2}}{MSE}\right)$$

where the mean square error (MSE) is defined as

(15) $$MSE={\left\|G-\hat{G}\right\|}_{2}^{2}/\left(m\times n\right)$$

Table 4 presents the PSNR value for various images under different measurement matrices with the measurement number of 120. Our proposed measurement matrix achieved the highest peak signal-to-noise ratio (PSNR) among the compared measurement matrices.

In addition to the PSNR, we employed the structural similarity measure (SSIM) as a complementary evaluation metric. As shown in Table 5, the results indicate that with a measurement number of 120, our matrix achieved the highest SSIM value compared to the other measurement matrices. This indicates that our proposed measurement matrix outperformed other measurement matrices in preserving the structural details.

**Figure 6:**
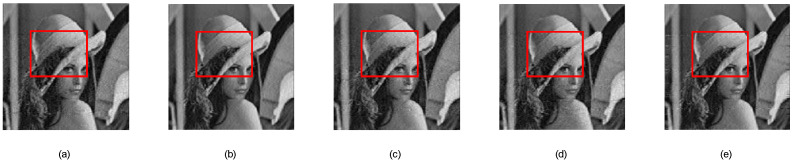
Reconstructions of Lena: (**a**) Gaussian random measurement matrix, (**b**) our proposed measurement matrix, (**c**) random binary measurement matrix, (**d**) random symmetric measurement matrix, and (**e**) bipartite graph measurement matrix.

**Figure 7:**
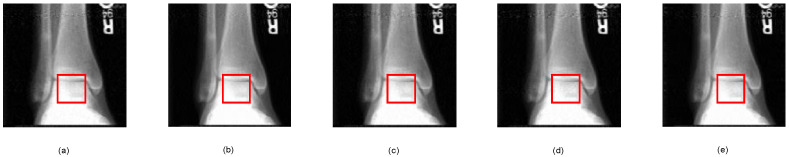
Reconstructions of Bone2: (**a**) Gaussian random measurement matrix, (**b**) our proposed measurement matrix, (**c**) random binary measurement matrix, (**d**) random symmetric measurement matrix, and (**e**) bipartite graph measurement matrix.

**Figure 8:**
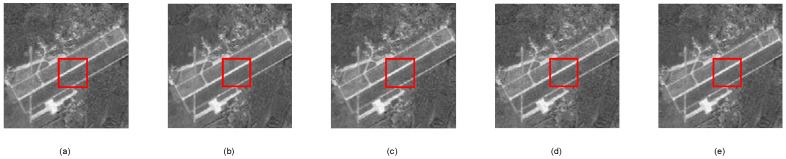
Reconstructions of Aerial image2. (**a**) Gaussian random measurement matrix, (**b**) our proposed measurement matrix, (**c**) random binary measurement matrix, (**d**) random symmetric measurement matrix, and (**e**) bipartite graph measurement matrix.

**Figure 9:**
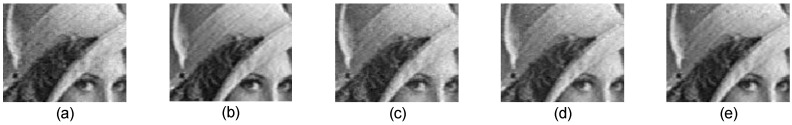
Details of reconstructed Lena: (**a**) Gaussian random measurement matrix, (**b**) our proposed measurement matrix, (**c**) random binary measurement matrix, (**d**) random symmetric measurement matrix, and (**e**) bipartite graph measurement matrix.

**Figure 10:**
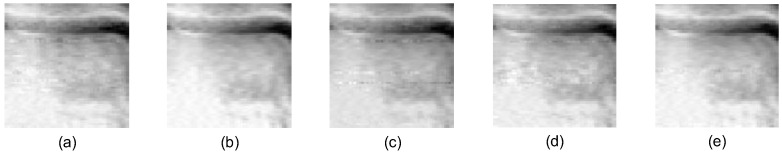
Details of reconstructed Bone2: (**a**) Gaussian random measurement matrix, (**b**) our proposed measurement matrix, (**c**) random binary measurement matrix, (**d**) random symmetric measurement matrix, and (**e**) bipartite graph measurement matrix.

**Figure 11:**
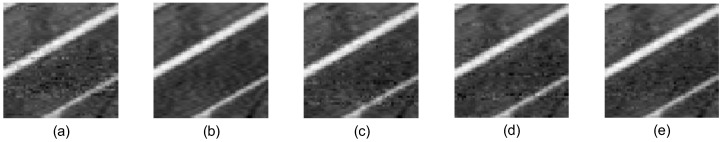
Details of reconstructed Aerial image2: (**a**) Gaussian random measurement matrix, (**b**) our proposed measurement matrix, (**c**) random binary measurement matrix, (**d**) random symmetric measurement matrix, and (**e**) bipartite graph measurement matrix.

Figure 6, Figure 7 and Figure 8 individually show the visually reconstructed results of the images “Lena”, “Bone2”, and “Aerial Image2.” The details of the areas within the red boxes are shown in Figure 9, Figure 10 and Figure 11, respectively. For the Lena and Aerial Image2 images, the block effects in the reconstructed images using our proposed measurement matrix were significantly reduced, resulting in smoother images. In the Bone2 image, our matrix effectively minimized artifacts that were present with other matrices, demonstrating its superior performance in preserving intricate textures and details.

## 5. Conclusions

We propose a simple and adaptable deterministic sparse measurement matrix utilizing the Legendre sequence. Empirical analysis illustrates that its phase transition outperforms both random and bipartite graph measurement matrices. Additionally, the matrix exhibits favorable practical features. The simulation results confirm that it surpasses random and deterministic bipartite graph measurement matrices in reconstruction quality. In future work, we will conduct a more in-depth investigation into the pseudo-random properties of sequences and explore alternative construction methods for deterministic measurement matrices.

## Figures and Tables

**Table 1:** Prime numbers in the form of 4t−1 between 100 and 1000.

103	107	127	131	139	151	163	167	179	191
199	211	223	227	239	251	263	271	283	307
311	331	347	359	367	379	383	419	431	439
443	463	467	479	487	491	499	503	523	547
563	571	587	599	607	619	631	643	647	659
683	691	719	727	739	743	751	787	811	823
827	839	859	863	883	887	907	911	919	947
967	971	983	991						

**Table 3:** Recovery SNRs of various measurement matrices in noisy case with k=100 and N=431.

	Measurement Number	This Paper	Gaussian Random	Random Symmetric	Random Sparse	Bipartite Graph
Gaussian random sparse signals	220	7.77	7.62	7.66	7.69	7.76
	280	17.80	17.59	17.75	17.77	17.77
	340	20.03	19.79	19.99	19.98	20.00
	400	21.40	21.16	21.37	21.38	21.38
Uniform random sparse signals	220	−0.90	−0.91	−0.92	−0.92	−0.91
	280	1.62	1.47	1.47	1.47	1.49
	340	5.16	4.91	4.97	5.01	5.01
	400	10.28	10.01	10.13	10.12	10.19

**Table 4:** Recovery PSNRs of various images.

Images	This Paper	Gaussian Random	Random Symmetric	Random Sparse	Bipartite Graph
Lena	28.96	23.95	25.10	24.98	25.75
Baboon	27.48	23.81	24.13	24.00	24.94
Barbara	30.53	26.74	27.22	26.93	28.00
Cameraman	27.00	23.11	22.92	23.18	24.27
Goldhill	31.64	26.56	27.46	27.11	28.48
Peppers	30.39	26.14	26.67	26.10	26.98
Bone1	33.49	31.34	31.36	31.18	30.59
Bone2	34.08	29.13	30.77	29.88	31.80
Aerial image1	25.66	21.13	20.52	20.27	22.67
Aerial image2	29.85	24.93	25.29	25.81	26.83

**Table 5:** Recovery SSIM of various images.

Images	This Paper	Gaussian Random	Random Symmetric	Random Sparse	Bipartite Graph
Lena	0.842	0.723	0.757	0.731	0793
Baboon	0.835	0.719	0.723	0.723	0.763
Barbara	0.859	0.782	0.784	0.796	0.831
Cameraman	0.772	0.605	0.644	0.616	0.709
Goldhill	0.907	0.796	0.841	0.826	0.868
Peppers	0.870	0.765	0.808	0.793	0.823
Bone1	0.935	0.889	0.894	0.897	0.897
Bone2	0.910	0.830	0.871	0.850	0.885
Aerial image1	0.859	0.721	0.760	0.741	0.811
Aerial image2	0.840	0.638	0.712	0.693	0.772

## Data Availability

The data presented in this study are available on request from the corresponding author.
